# Post-traumatic stress disorder - are we doing all we can? A UK national-based survey

**DOI:** 10.1186/cc12475

**Published:** 2013-03-19

**Authors:** X Watson

**Affiliations:** 1 Ashford and St Peter's Hospital, Surrey, UK

## Introduction

Post-traumatic stress disorder (PTSD) is a well-recognised complication in patients discharged from the ICU. ICU clinics have been recommended to treat physical and psychosocial problems post discharge, with guidelines issued by the Department of Health UK [1]. Recent evidence has advocated the use of patient diaries to reduce the incidence of new-onset PTSD [2]. Family support groups may also play a pivotal role [3]. We performed a national survey via the Intensive Care Society (ICS) to determine the provision of follow-up clinics, patient diary services and family support groups.

## Methods

An online structured questionnaire was sent via the ICS to all ICU linkmen at 124 hospitals in the UK. Responses were received from 77 ICUs, a 62% response rate.

## Results

Out of 77 ICUs, 37 (48%) run a follow-up clinic. The majority of clinics (51%) only invite patients who have been admitted for more than 3 to 4 days. Only 10 clinics (30%) receive funding from the ICU budget or PCT, with the majority (67%) receiving no funding at all. Only 44% (34) of ICUs use patient diaries, mostly as a nonfunded service (68%). Additionally, 91% of ICUs do not run family support groups; from the minority that do, these are mostly held quarterly and are largely not funded (55%).

## Conclusion

This survey demonstrates that the provision of ICU clinics in the UK is not well established, with only 48% currently providing a regular service. Currently 67% of clinics are not funded and further resources should be employed so this service becomes an integral part of the ICU pathway. Despite recent evidence demonstrating that diaries reduce new-onset PTSD, only 44% of ICUs in our study provide this service. Ninety-one per cent of ICUs do not provide family support groups; similarly, it appears that financial constraints are the limiting factor.
